# Protecting RNA quality for spatial transcriptomics while improving immunofluorescent staining quality

**DOI:** 10.3389/fnins.2023.1198154

**Published:** 2023-05-18

**Authors:** Nina Hahn, Martin Bens, Marin Kempfer, Christin Reißig, Lars Schmidl, Christian Geis

**Affiliations:** ^1^Section of Translational Neuroimmunology, Department of Neurology, Jena University Hospital, Jena, Germany; ^2^Center for Sepsis Control and Care, Jena University Hospital, Jena, Germany; ^3^Leibniz Institute on Aging – Fritz Lipmann Institute (FLI), Jena, Germany

**Keywords:** spatial transcriptomics, Visium spatial, mouse brain transcriptome, RNA quality, RNA protection, immunofluorescent staining, methanol fixation

## Abstract

In comparison to bulk sequencing or single cell sequencing, spatial transcriptomics preserves the spatial information in tissue slices and can even be mapped to immunofluorescent stainings, allowing translation of gene expression information into their spatial context. This enables to unravel complex interactions of neighboring cells or to link cell morphology to transcriptome data. The 10× Genomics Visium platform offers to combine spatial transcriptomics with immunofluorescent staining of cryo-sectioned tissue slices. We applied this technique to fresh frozen mouse brain slices and developed a protocol that still protects RNA quality while improving buffers for immunofluorescent staining. We investigated the impact of various parameters, including fixation time and buffer composition, on RNA quality and antibody binding. Here, we propose an improved version of the manufacturer protocol, which does not alter RNA quality and facilitates the use of multiple additional antibodies that were not compatible with the manufacturer protocol before. Finally, we discuss the influence of various staining parameters, which contribute to the development of application specific staining protocols.

## 1. Introduction

The understanding of mechanisms underlying neurodegenerative diseases has been fueled by the development of new methods unraveling pathological alteration in the transcriptome. Starting from bulk ribonucleic acid (RNA) sequencing, which identifies the average expression across cell populations, RNA sequencing developed to single cell resolution. This enables the discrimination between individual cell types within a population and even between cell states. However, single cell RNA sequencing loses any information about spatial relationships or cell morphology, which are often necessary for in-depth investigation of complex interactions between cells.

Spatial information on gene expression can be precisely obtained by fluorescent *in situ* hybridization (FISH) based techniques (Itzkovitz and van Oudenaarden, 2011; Shakoori, 2017). There, tissue slices or whole mount preparations are incubated with fluorescently labeled probes to detect transcripts of interest. This approach offers a high sensitivity with the ability to resolve single transcripts (Femino et al., 1998; Itzkovitz and van Oudenaarden, 2011). However, FISH has a substantial drawback. The selection of probes limits insights to a pre-defined set of marker genes and thereby does not represent the whole transcriptome. Recently developed spatial transcriptomic platforms with spatial barcode technologies can preserve tissue architecture information while enabling whole transcriptome sequencing. So far, various platforms have been developed, each with certain limitations. Stereo-Seq provides excellent spatial resolution (spot size 220 nm) and is applicable to large tissue samples (capture area 200 mm^2^) such as an entire macaque brain (Chen et al., 2022). Nevertheless, drawbacks of such high-resolution include a high effort for sequencing, proper restriction of messenger RNA diffusion and decreased sensitivity. In contrast, the 10× Genomics Visium platform allows the investigation of 10 μm thick tissue sections with a size of 6.5 × 6.5 mm (Ståhl et al., 2016). Tissue sections are placed on a microscope slide that is equipped with 5,000 barcoded spots (diameter 55 μm) which capture polyadenylated transcripts after tissue permeabilization. This approach leads to a lower spatial resolution of 1–10 cells per spot but offers the great opportunity to perform immunostaining and transcriptome analysis within the same tissue section. Thus, not only information about tissue architecture is preserved but also cell morphology. To improve the spatial resolution of 1–10 cells per spot, the Visium platform can be combined with single nuclei sequencing using an adjacent tissue slice. High-resolution single nuclei data can then be mapped back to the spatial transcriptome data using deconvolution algorithms (Elosua-Bayes et al., 2021; Cable et al., 2022; Kleshchevnikov et al., 2022; Lopez et al., 2022) to infer the cell composition of each spot.

Here, we tested various parameters including tissue fixation, staining buffer composition and RNA protecting agents that influence RNA quality and antibody binding, thus providing a toolbox to optimize immunofluorescent staining protocols for spatial transcriptomics. We applied our optimized protocol to fresh frozen mouse brain slices and used the 10× Genomics Visium platform to analyze the transcriptome.

## 2. Materials and methods

### 2.1. Animals and tissue dissection

All animal experiments have been approved by the Thuringian state authorities (authorization twz08-2020 and UKJ-18-026). We used male C57BL/6 J mice of our own breeding facility (service center for small rodents, Jena, University Hospital) at the age of 10–16 weeks. Animals were housed under controlled day/night (12 h/12 h) conditions at room temperature (23 ± 1°C, 30%–60% environmental humidity) and received a standard diet and water *ad libitum*.

For tissue collection, mice were deeply anesthetized with isoflurane, transcardially perfused with 25 mL of phosphate buffered saline (PBS) for 5 min. The brain was dissected and snap frozen for 1 min in isopentane placed in a nitrogen bath prior to OCT embedding and storage at −80°C. Tissue was sliced to 10 μm this sections using a cryostat (Leica CM3050 S, Nussloch, Germany) and stored up to 4 weeks in a sealed container with a moisture absorbing pad at −80°C. To measure RNA integrity, 10 brain tissue sections were collected in a tube for RNA extraction with QIAzol lysis reagent (Qiagen, #79306) according to the user manual. The RNA integrity was measured with an Agilent 2100 Bioanalyzer (Santa Clara, United States) and the calculated RIN value was 9.4.

### 2.2. Immunostaining and imaging

Microscope slides were removed from −80°C, placed on carbon dioxide snow and processed quickly to protect RNA quality. Slides were dried at 37°C for 1 minute using a thermocycler (TOne 96 G, Biometra, Jena, Germany) equipped with the 10× Genomics thermocycler adaptor and subsequently placed into ice-cold methanol or other organic solvent (compare Table 1). Fixation duration ranged from dipping up to 30 min at −20°C, as indicated. In addition, fixation with ethanol and acetone was tested (compare Table 1). After fixation, OCT was removed with the aid of forceps and the microscope slide was placed into the slide cassette. The reference stainings are performed according to the protocol demonstrated by 10× Genomics for methanol fixation and immunofluorescent staining, Rev. B (#CG000312). In our modified protocol, slices were washed twice prior to 20 min blocking and 30 min primary antibody incubation at room temperature. Then, slices were washed three times with wash buffer, 15 min incubated with the secondary antibodies and DAPI, washed four times and finally 20 times rinsed in 3× saline-sodium citrate (SSC) buffer. We tested various blocking, wash and antibody binding buffers that are summarized in Table 2. The mounting medium consisted of 170 μL sterile glycerol, 20 μL RNAse inhibitor (RNAsin, New England Biolabs, #M0314L) and 10 μL water.

**Table 1:** Evaluation of tissue fixation with organic solvents.

Fixative	Fixation time	Staining quality
Methanol	Dipping	−
Methanol	5 min	++
Methanol	10 min	+
Methanol	20 min	No staining
Methanol	30 min	No staining
Ethanol	5 min	−
Ethanol	30 min	No staining
Acetone	5 min	No staining
Acetone	10 min	No staining
Acetone:Methanol 1:1	5 min	No staining

The staining quality was exemplary assessed on the basis of TMEM119 and DAPI staining. –, very poor staining; +, good staining; ++, very good staining.

**Table 2:** Summary of tested washing, blocking and antibody binding buffers.

Solution	pH value
1× PBS	pH 7.4
1× PBS	pH 7 Adjusted from pH 7.4
3× SSC	pH 7
3× SSC	pH 7.4 Adjusted from pH 7
BSA Merck 10%	pH 6.5
BSA MACS 10%	pH 7.2

Slides were imaged with a LSM900 (Carl Zeiss Microscopy GmbH, Oberkochen, Germany) equipped with an EC “Plan-Neofluar” 10×/0.30 M27 objective (Carl Zeiss Microscopy GmbH, Oberkochen, Germany) and Zen Blue Software (Version 3.1, Carl Zeiss Microscopy GmbH, Oberkochen, Germany). Tissue optimization slides were imaged as one tile region to ensure comparison. The overlap between tiles was set to 10% and stitching was performed with standard settings without shading correction and saved as tiff files. Fluorescently labeled cDNA on tissue optimization slides was imaged with the texas red filter (Zeiss #45; 40 ms exposure) while immunostainings on gene expression slides were imaged with a DAPI (Zeiss #49; 5 ms exposure), texas red (Zeiss #45; 150 ms exposure), green fluorescent protein (Zeiss #38; 80 ms exposure) and a Cy5 filter (Zeiss #50; 300 ms exposure).

### 2.3. Antibodies

Used antibodies are listed in Table 3.

**Table 3:** Result summary of tested antibodies in our optimized protocol.

Target	Cell type marker	Host	Supplier	Product ID	RRID	Dilution; concentration	Usability
Bassoon	Presynapse	gp	Synaptic systems	141 004	AB_2290619	1:500 from antiserum	Yes
Gephyrin	Inhibitory postsynapse	ms	Synaptic systems	147 011	AB_887717	1:100; 10 μg/mL	No
GFAP	Astrocyte	ms	Biotium	BNUM0789-50	Clone ASTRO/789	1:100; 10 μg/mL:	Yes
Homer1	Excitatory postsynapse	ch	Synaptic systems	160 006	AB_2631222	1:400; 2.5 μg/mL	Yes
HopE	Neural stem cells	ms	Santa Cruz	sc-398703	AB_2687966	1:50; 4 μg/mL	No
Mannose (CD206)	Border associated macrophages	rb	Abcam	ab64693	AB_1523910	1:250; 4 μg/mL	Yes
Map2	Neurons	ch	Synaptic systems	188 006	AB_2619881	1:500; 2 μg/mL	Yes
PSD95	Excitatory postsynapse	ms	StressMarq	SMC-122D	AB_2300386	1:500; 2 μg/mL	No
PU.1	Mikroglia	rb	Cell Signaling Technology	2258S	AB_2186909	1:25; 6 μg/mL	Yes
Sox2	Neural stem cells	rb	Abcam	ab97959	AB_2341193	1:100; 10 μg/mL	No
Sox2	Neural stemm cells	ms	Abcam	ab79351	AB_10710406	1.100; 10 μg/mL	No
TMEM119	Mikroglia	gp	Synaptic systems	400 004	AB_2744645	1:100 from antiserum	Yes
VGAT	Inhibitory presynapse	gp	Synaptic systems	131 004	AB_887873	1:500 from antiserum	Yes
VGlut1	Excitatory presynapse	gp	Synaptic systems	135 304	AB_887878	1:750 from antiserum	Yes
VGlut1	Excitatory presynapse	rb	Synaptic systems	135 303	AB_887875	1:500; 2 μg/mL	Yes

Lyophilized antibodies were reconstituted according to the data sheet. ch, chicken; gp, guinea pig; ms, mouse; rb rabbit.

### 2.4. Tissue permeabilization and fluorescently labeled cDNA synthesis

Tissue permeabilization was conducted according to the user manual of the tissue optimization kit (#000193, 10× Genomics, Pleasanton, CA, United States). First, several permeabilization times were tested ranging from 30 min to 6 min, selecting 8 min as the most appropriate permeabilization time for following experiments (Supplementary Figure S1). In order to test the RNA preserving capability of various staining and washing buffers, slides from the tissue optimization kit were used and fluorescently labeled complementary deoxyribonucleic acid (cDNA) was synthesized according to the user manual. The fluorescence intensity indicates the quality of RNA used for cDNA synthesis and was assessed qualitatively. Comparisons between tissues were performed between with adjacent tissue slices on the identical microscope slide.

### 2.5. Visium spatial transcriptomics library preparation and sequencing

cDNA synthesis, second strand synthesis and cDNA amplification were performed according to the Visium spatial gene expression user guide CG000239, Rev. D (#1000187, 10× Genomics, Pleasanton, CA, United States). The cycle number for cDNA amplification was determined by qPCR using a Corbett Rotor-Gene 6000 thermocycler. Finally, cDNA was cleaned up by SPRIselect (#B23317, BeckmannCoulter, Krefeld, Germany) and checked for quality and quantity using an Agilent 2100 Bioanalyzer instrument and a high sensitivity DNA kit. The library was constructed using 10 μL of total cDNA following the user manual. Quantification and quality check of libraries was performed using the Agilent 2100 Bioanalyzer instrument and DNA 7500 kit. Libraries from each slide were pooled and each pool sequenced on NovaSeq 6000 using 100 cycle SP Reagent Kit v1.5 (Read1 = 28, Read2 = 90, Index1 and Index2 = 10). Base calling was performed using bcl2fastq (v2.20.0.422).

### 2.6. Visium data procession and quality control

Manual fiducial alignment and tissue outlining was performed using Loupe Browser (v6.1.0). Samples were processed with spaceranger (1.3.1) based on mouse reference genome mm10 (reference package refdata-gex-mm10-2020-A). Spaceranger data was further analyzed with R (version 4.2.2) and R Studio (version 2022.02.2) using the Seurat package (Hao et al., 2021). For visualization, dplyr, ggplot2, patchwork, hdf5r and viridis packages were used (Wickham, 2016; Garnier et al., 2021; Hoefling and Annau, 2022; Pedersen, 2022; Wickham et al., 2022). As reference, two mouse brain datasets from 10× Genomics were integrated. Similar to our samples, both are from adult mice, coronal 10 μM thick coronal cryosection. The dataset “10×_HE” was retrieved from https://www.10xgenomics.com/resources/datasets/mouse-brain-section-coronal-1-standard and collected from hematoxylin and eosin stained tissue. The dataset “10x_IF” was retrieved from https://www.10xgenomics.com/resources/datasets/adult-mouse-brain-section-2-coronal-stains-dapi-anti-gfap-anti-neu-n-1-standard-1-1-0 and collected from DAPI, anti-GFAP and anti-NeuN stained tissue. Statistical analysis using a pairwise permutation test was performed with the aid of the coin and rcompanion packages (Hothorn et al., 2006, 2008; Mangiafico, 2023).

## 3. Results and discussion

### 3.1. Tissue fixation with organic solvents

Along with tissue collection, tissue fixation is one of the main critical steps for immunostainings and transcriptome analysis since it helps to preserve both, protein and RNA quality. The protocol demonstrated by 10× Genomics suggests 30 min fixation with ice-cold methanol, however, this does not lead to successful staining with all our tested antibodies. We found that reducing the fixation time to 5 min leads to optimal staining results on 10 μm thick mouse brain cryosections (Table 1) while preserving RNA quality properly. In addition, reducing the fixation time has the benefit of reducing the risk of RNA leakage (Esser et al., 1995). Besides methanol, we tested ethanol and acetone as organic solvents. However, methanol fixation outperformed both. We could further improve the immunostaining by introducing two additional washing steps after methanol fixation to rehydrate the sample, which markedly recovers specific antibody binding.

Most antibodies validated for immunostainings were tested on native or paraformaldehyde (PFA) fixed tissue and hence do not recognize epitopes after fixation with organic solvents. For spatial transcriptomics, PFA fixation is less suited, since PFA leads to RNA degradation as well as RNA modification (Ben-Ezra et al., 1991; Evers et al., 2011). Organic solvents such as methanol dehydrate the sample leading to precipitation of proteins and affecting RNA quality to a lesser extent compared to PFA (Ben-Ezra et al., 1991; Su et al., 2004). Due to precipitation, epitopes change their conformation and are often not recognized by antibodies that have been validated on PFA fixed tissue. However, we found that antibodies validated for flow cytometry applications are often better suited for the Visium platform in combination with immunofluorescence staining. Cells for flow cytometric assays are typically fixed with ice-cold ethanol, which may explain this phenomenon.

### 3.2. Blocking and antibody binding buffer composition

**Figure 1:**
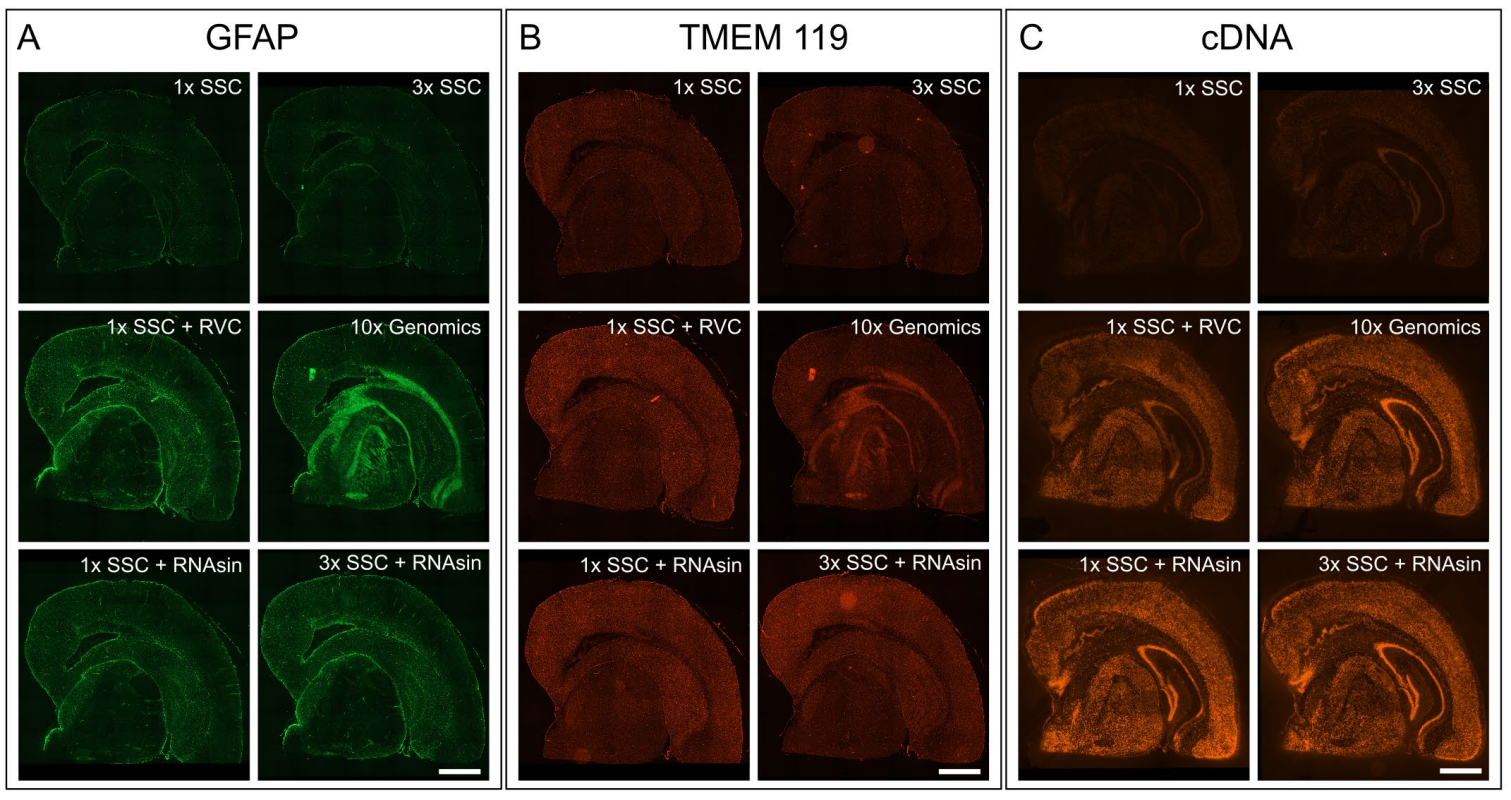
Impact of RVC, SSC buffer and RNAse inhibitor (RNAsin) on GFAP astrocyte marker staining **(A)**, TMEM119 microglia marker staining **(B)** and RNA quality protection based on fluorescently labeled cDNA **(C)**. The demonstrated 10× Genomics protocol was used as a reference (middle right). Scale bar 1 mm.

The blocking buffer introduced by the manufacturer protocol is composed of 3× SSC buffer, 2% bovine serum albumin (BSA), 0.1% Triton X-100, 1 U/μL RNase inhibitor, 20 mM Ribonucleoside Vanadyl complex (RVC) and 14 μg/mL TruStain FcX (#101319, BioLegend, San Diego, United States). For the primary antibody binding solution, 270 U RNase inhibitor are added to the blocking buffer. With all tested antibodies this protocol leads to no or very weak staining, high background and blurred unspecific fluorescent signals (Figures 1A,B; middle right). Hence, we started to decipher the respective characteristics of each component to control for its advantages as well as disadvantages during blocking and antibody binding.

First, we focused on components that are not involved in RNA protection but can interfere with immunostaining. Triton X-100 permeabilizes the cell membrane but is also known to interfere with immunostaining of membrane-bound proteins (Oliver and Jamur, 1984; Hobro and Smith, 2017). Additional permeabilization with Triton X-100 was not necessary for staining for intracellular antigens, as methanol already permeabilises membranes (Oliver and Jamur, 1984; Hobro and Smith, 2017), so we left it out. In addition, we excluded TruStain FcX which blocks unwanted Fc receptor binding. Furthermore, we increased the BSA concentration from 2% to 10% and thereby noticeably improved the stainings and reduced background.

### 3.3. RVC interferes with immunostaining and is inactivated by SSC buffer

It is crucial to protect the RNA for downstream transcriptome analysis during the course of immunostaining and imaging which last approximately 4 h in total and is performed at room temperature. To avoid RNA degradation by ribonucleases, 10× Genomics recommends adding 20 mM RVC in blocking, wash and antibody binding buffer. RVC is an efficient and inexpensive inhibitor of RNAse (Shieh et al., 2018). However, we found that RVC leads to blurred, unspecific fluorescent signals and prevents antibodies from specific binding, when applied according to the manufacturer protocol (Figures 1A,B; middle right). Even reducing the RVC concentration from 20 mM to 10 mM as recommended by the RVC product manual, does not improve immunofluorescent staining. We needed to reduce the concentration of RVC further to 1 mM to exclude interference with antibody binding (Figures 1A,B; middle left). In line with previous results that showed RNA protective behaviour of RVC at 0.2 mM (Shieh et al., 2018), we observe RNA protection using 1 U/mL RNAsin and 1 mM RVC, however markedly less than 20 mM RVC (Figure 1C; middle left).

**Figure 2:**
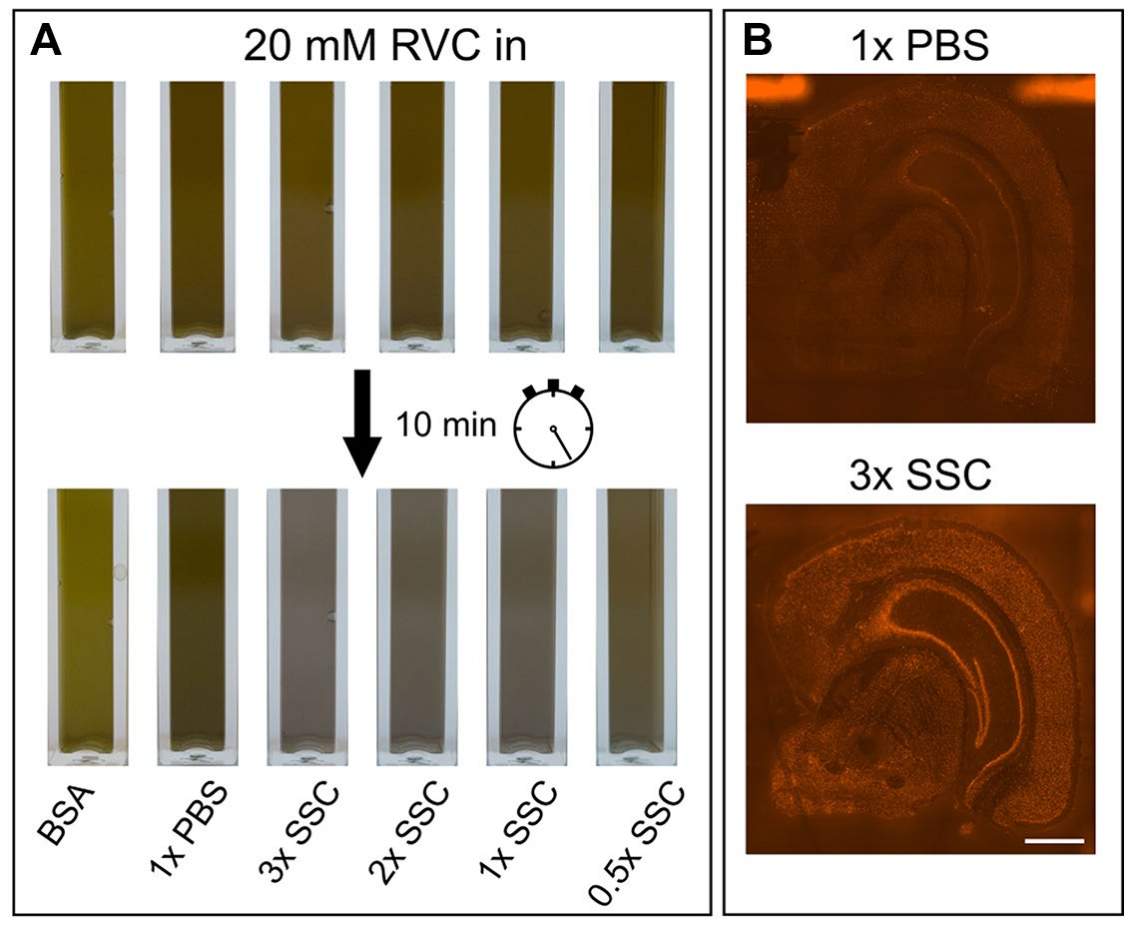
Influence of PBS, SSC and RVC in wash buffer. **(A)** Color change of RVC after 10 min diluted in BSA, PBS and various SSC buffer concentrations. Forest green indicates active RVC, black color inactive RVC. **(B)** Effect of 1× PBS and 3× SSC on RNA quality measured on the basis of fluorescently labeled cDNA. 3× SSC protects RNA better against degradation than 1× PBS. Scale bar 1 mm.

RVC is very sensitive to oxidation and dissociation. After reconstitution, it should have a brilliant forest green color. When dissociated, it turns black and loses its capability as an inhibitor of RNAses (Shankar and Ramasarma, 1993). Figure 2A shows that addition of RVC to SSC buffer leads to a change of color from forest green to black after 10 min, even if the SSC buffer is diluted to a concentration of 0.5X. In addition, a black precipitate was noticed. According to the user manual, RVC should not be used in the presence of ethylenediaminetetraacetic acid (EDTA), which leads to dissociation of the complex. EDTA and citrate are both chelating agents. Thus, sodium citrate in SSC buffer will lead to dissociation and inactivation of RVC as well. This is in line with previous reports that 2× SSC buffer reduces the effective lifetime of RVC in MERFISH stainings (Moffitt et al., 2016). Altogether, this indicates that RVC is inactivated by SSC buffer and should not provide substantial RNA protection in the buffers used for immunostaining suggested by the manufacturer. Interfering with the immunostaining whilst providing only limited protection of RNA quality, we excluded RVC from our staining buffers. Instead, we included RNAse inhibitor in all of our buffers with a final concentration of 1,000 U/mL. Indeed, replacement of RVC with RNAse inhibitor does not interfere with antibody binding while reliably protecting RNA quality (Figures 1A–C; bottom row). In addition, the used RNAse inhibitor is stable at room temperature providing a robust protection during immunostaining as well as during imaging steps. However, in comparison to RVC, RNAse inhibitor is more expensive and increases the cost for each microliter of wash buffer 80–90 times.

### 3.4. Increasing antibody binding without harming RNA quality

The main component of the wash buffer for immunostaining is 3× SSC. We found that increasing concentration of SSC in the antibody binding buffer decreases antibody binding (Figures 1A,B; upper row). Substituting 3× SSC by 1× PBS, which is the most common buffer for immunostainings, remarkably increased specific antibody binding. However, this dramatically decreased RNA quality (Figure 2B).

Lowering the pH value and increasing the ionic strength are common approaches to adjust binding properties in affinity chromatography or co-immunoprecipitation (Roberts et al., 2015). 3× SSC (pH 7.0 at room temperature) and PBS (pH 7.4 at room temperature) buffer differ in both, pH and ionic strength. Decreasing the pH of PBS to 7 as present in SSC buffer, prevented antibody binding. In contrast, increasing the pH to 7.4 in 3× SSC buffer did not promote antibody binding. Thus, a lower pH value decreases antibody binding but is not the only reason why 3× SSC interferes with proper immunostaining. 3× SSC buffer is typically used in FISH or northern blotting protocols, since its higher salt concentration supports RNA integrity. The salt concentration in 3× SSC buffer is higher than in 1× PBS buffer, hence, we tested as well 1× SSC buffer (Figures 1A–C; left column) to evaluate the effect of ionic strength.

Usually, antibodies for immunostainings are selected with antibody binding buffers based on PBS (pH 7.4) and TBS (pH 7.6). Thus, antibodies for immunostaining might be selected for complement-determining regions (CDRs) that form strong bounds at pH 7.4–7.6 but only weak bounds at pH 7. Depending on the CDR composition, antibodies are susceptive to pH change. Specifically, CDRs composed of aspartic acid are vulnerable to variation in pH (Psimadas et al., 2012). In addition, protein–protein interaction of immunoglobulins is more attractive with an increasing pH (Roberts et al., 2015). This effect might be particularly substantial since epitopes might suffer from slight conformational changes due to methanol fixation challenging antibody to antigen binding anyway.

Due to the effect of pH on antibody binding, we carefully tested the pH of BSA solutions suggested in the demonstrated protocols. BSA from Merck (pH 6.9; #126615-25ML) prevents antibody binding, while BSA MACS (pH 7.4;Miltenyi Biotec, #130-091-376) leads to specific staining with low background. This is in line, with our previous results, showing pH 7 as inappropriate for antibody binding.

We tested whether a lower pH and a higher salt concentration only affects antibody binding itself or even the binding persistence of an already bound antibody. While 3× SSC in the antibody binding buffer prevents antibody binding, 3× SSC in the washing buffer does not affect immunostaining quality. Consequently, we diluted the antibodies in 10% BSA blocking buffer, not using SSC buffer during the binding phase, but keeping it in the wash buffer to protect RNA. Wash buffer composed of 3× SSC (Figure 1C; upper right) shows a stronger protective effect on RNA quality compared to wash buffer with 1× SSC (Figure 1C; upper left).

In summary, our optimized protocol reduces methanol fixation time to 5 min, omits TruStain FcX, Triton X-100 and RVC but adds RNAse inhibitor to all buffers with a concentration of 1,000 U/mL. In addition, we inserted an additional washing step after methanol fixation to rehydrate the tissue samples and replaced 3× SSC containing blocking and antibody binding buffer by 10% BSA. Antibodies targeting various cell types and that are usable with our protocol are shown in Table 3 and Supplementary Figure S4. Further stainings with antibodies that were not compatible are shown in Supplementary Figure S5.

### 3.5. Staining protocol optimization does not affect the quality of spatial transcriptome data

**Figure 3:**
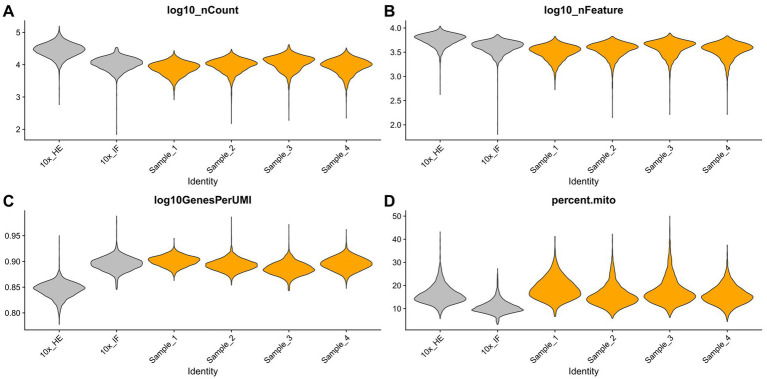
Comparison of four samples stained with our optimized IF staining protocol (orange) to 10× Genomics reference data sets (HE stained and IF stained, grey). Violin plots summarize the respective transcript counts **(A)**, features **(B)**, the complexity (log10 genes per UMI) **(C)** and mitochondrial related transcript counts **(D)**.

**Figure 4:**
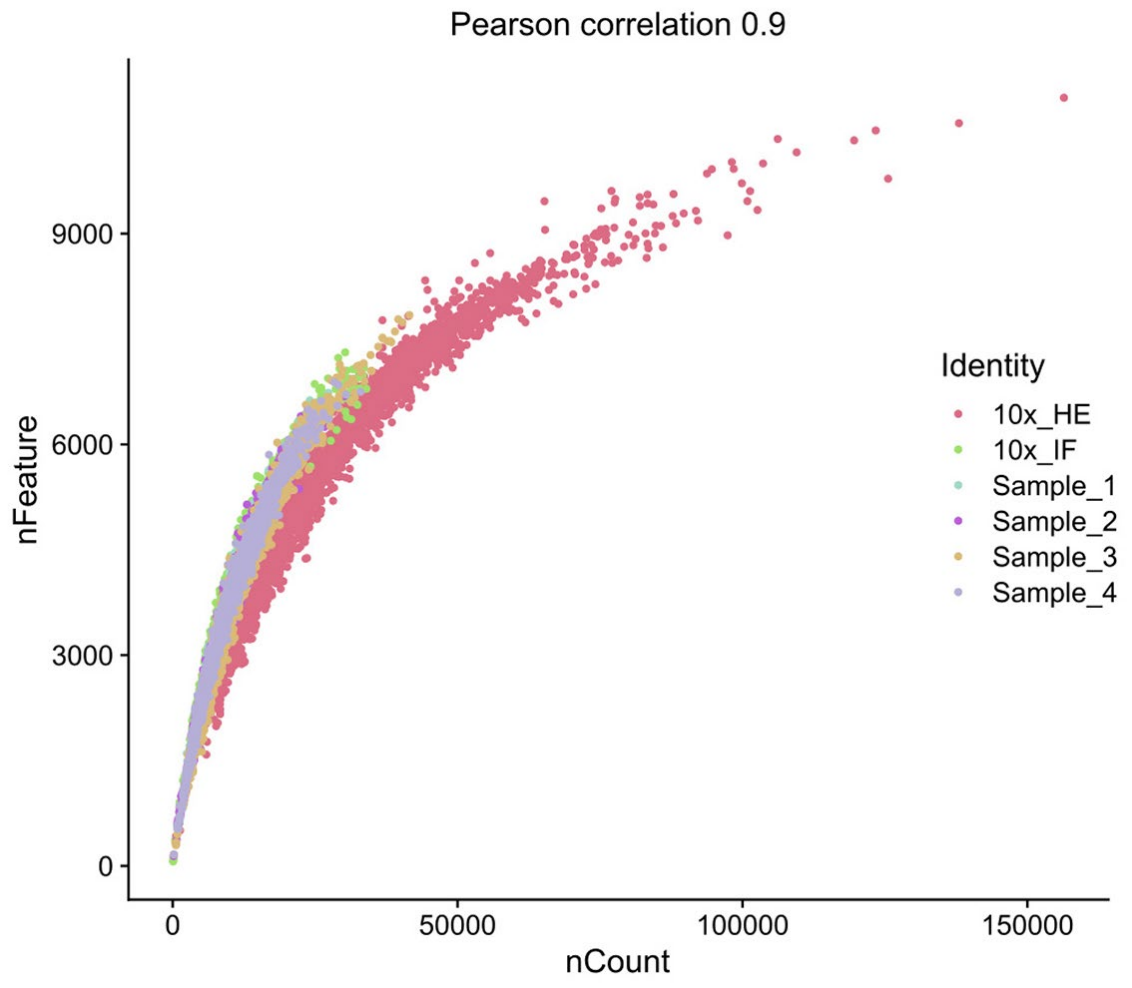
Scatterplot representing the correlation of nFeature and nCount.

Improving immunostaining must not worsen RNA integrity and consequently spatial transcriptome data quality. We sequenced libraries using the Visium platform from four samples and compared their quality to 10× Genomics mouse brain datasets (10×_HE and 10×_IF) as reference. While the dataset “10×_HE” was derived from hematoxylin and eosin (HE) stained tissue, the data set “10×_IF” was derived from tissue after immunofluorescent (IF) staining with DAPI, anti-GFAP and anti-NeuN according to the manufacturer demonstrated protocol for methanol fixation and IF staining. Figure 3A and Table 4 shows a similar number of log10 transcript counts per spot using our optimized protocol (minimum 2.18–2.91; median 3.90–4.09) compared to 10×_IF (minimum 1.84; median 4.04). Using HE stained tissue leads to higher log10 transcript counts in comparison to IF stained tissue (minimum 2.76, median 4.46). Figure 4 shows that the number of transcript counts (nCount) and number of detected genes (nFeature) per spot correlate well with another (Pearson coefficient: 0.9) and that IF stained samples are highly similar.

**Table 4:** Summary of main sequencing metrices.

Sample IDs	Minimum Log10Counts	Median Log10Counts	Minimum Log10Features	Median Log10Features	Minimum complexity	Median complexity
10×_HE	2.76	4.46	2.63	3.78	0.78	0.85
10×_IF	1.84	4.04	1.80	3.63	0.85	0.90
Sample_1	2.91	3.90	2.72	3.52	0.86	0.90
Sample_2	2.18	4.00	2.15	3.58	0.85	0.89
Sample_3	2.28	4.09	2.21	3.63	0.84	0.89
Sample_4	2.35	3.98	2.21	3.57	0.85	0.90

The number of detected log10 features per spot in our samples (median 3.52–3.63, Figure 3B) is similar compared to the 10×_IF stained sample (median 3.63). In both, log10 transcript counts and log10 feature, the maximum variation between the 10×_IF reference sample and our samples is lower than the variation between 10×_HE stained and 10×_IF stained tissue. Comparing the minimum and median using a pairwise permutation test, does not detect any significant difference (*p* > 0.05). The number of total detected genes in IF stained tissue is similar and varies between 19,812 and 20,933 genes (Supplementary Table S1). The complexity (log10genesPerUMI) is lowest in the 10×_HE sample (minimum 0.78, median 0.85) and higher in IF stained samples (minimum 0.84, median 0.89–0.90) (Figure 3C; Table 4) with an even distribution throughout the tissue (Supplementary Figure S2). Overall, our staining protocol, which improves antibody binding, does not negatively affect RNA quality and sequencing thereby presenting an optimized alternative. Deviations between 10×_IF and our samples are lower than the deviation between 10×_HE and 10×_IF samples. A better performance of 10×_HE samples might be due to a markedly lower tissue staining time (approx. 30 min) compared to IF stained tissue (approx. 3 h). Mitochondrial counts are slightly higher in our samples (median 14.71–18.09) compared to 10× samples (median 15.33 and 10.25; Figure 3D). Supplementary Figure S3 shows that spots with higher mitochondrial counts are distributed along the tissue border indicating damage during tissue dissection and being independent from the staining protocol.

In summary, we systematically investigated the manufacturer immunostaining protocol for the Visium platform. The detailed knowledge, which characteristics of the buffer components affect antibody binding and RNA quality, helped to optimize the staining protocol that we propose for 10 μm mouse brain section. Our protocol preserves epitopes better and enhances antibody binding while it still protects RNA quality for downstream transcriptome analysis. In addition, we present suitable antibodies for often-used markers for several brain cell types and synaptic structures (Table 3). Overall, the findings presented here regarding the impact of fixatives and immunostaining buffers on RNA quality are as well crucial for optimizing protocols for other spatial transcriptomic platforms independent from the Visium platform.

## Data availability statement

The datasets presented in this study can be found in online repositories. The names of the repository/repositories and accession number(s) can be found at: https://www.ncbi.nlm.nih.gov/geo/, GSE228891.

## Ethics statement

The animal study was reviewed and approved by Thueringer Landesamt fuer Lebensmittelsicherheit und Verbraucherschutz.

## Author contributions

NH and CG: conceptualization and funding acquisition. NH, MB, and CR: methodology. NH, MB, MK, CR, and LS: investigation. CG: resources. NH and MB: data curation. NH: writing—original draft, supervision, and project administration. NH, MB, and CG: writing—review and editing. NH and MK: visualization. All authors contributed to the article and approved the submitted version.

## Funding

The study was funded by the Friedrich-Schiller-University program IMPULSEproject (IP2021-06 to NH), the Center for Sepsis Control and Care (CSCC; to NH and CG), and the Hermann and Lilly Schilling foundation (to CG). We acknowledge support by the German Research Foundation Project-No. 512648189 and the Open Access Publication Fund of the Thueringer Universitaets- und Landesbibliothek Jena.

## Conflict of interest

The authors declare that the research was conducted in the absence of any commercial or financial relationships that could be construed as a potential conflict of interest.

## Publisher’s note

All claims expressed in this article are solely those of the authors and do not necessarily represent those of their affiliated organizations, or those of the publisher, the editors and the reviewers. Any product that may be evaluated in this article, or claim that may be made by its manufacturer, is not guaranteed or endorsed by the publisher.
