# Supplementary material for: Protecting RNA quality for spatial transcriptomics while improving immunofluorescent staining quality
Source: Front Neurosci. 2023 May 18;17:1198154. doi: 10.3389/fnins.2023.1198154 (PMC10234422; doi:10.3389/fnins.2023.1198154)

Supplementary Figure 1: Tissue optimization assay to evaluate permeabilization times from 3 min to 30 min. Optimal permeabilization time was determined between 6 min and 12 min and was set to 8 min. Scale bar 1mm.

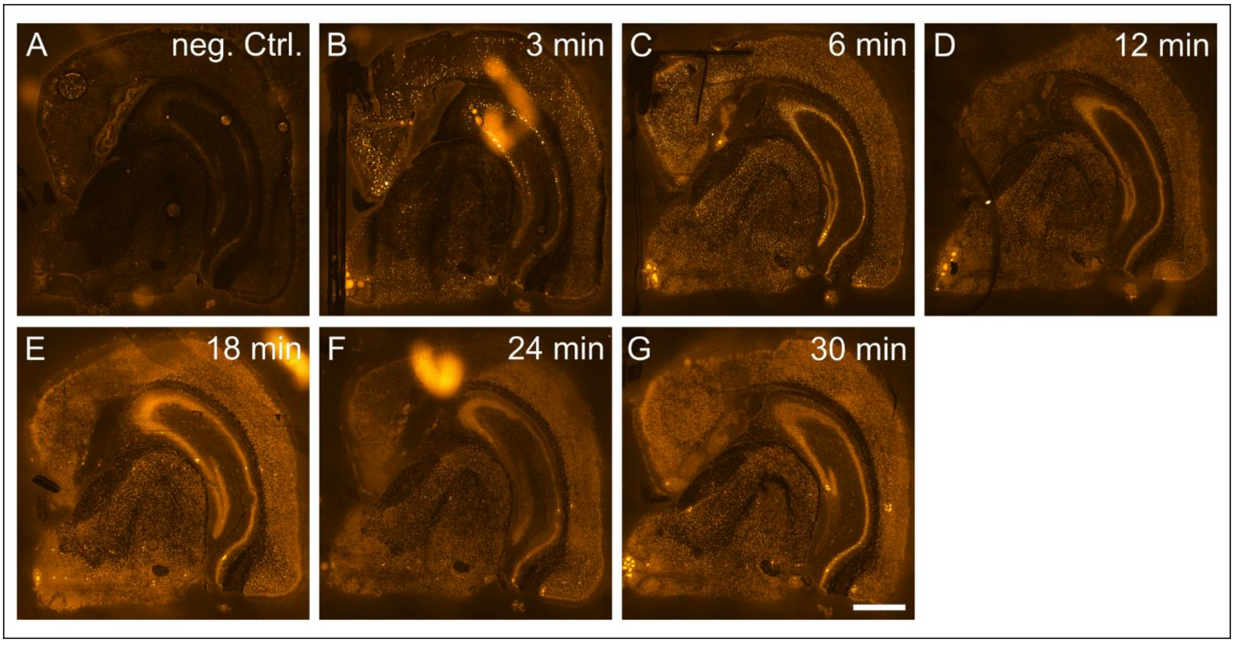

Supplement: Supplementary file 1 [file Data_Sheet_1.PDF]
