# Supplementary material for: Protecting RNA quality for spatial transcriptomics while improving immunofluorescent staining quality
Source: Front Neurosci. 2023 May 18;17:1198154. doi: 10.3389/fnins.2023.1198154 (PMC10234422; doi:10.3389/fnins.2023.1198154)

Supplementary Figure 3: Spatial plots showing the percentage of mitochondrial gene related counts of each spot. Samples from 10x genomics (10x_HE and 10x_IF) were used as reference.

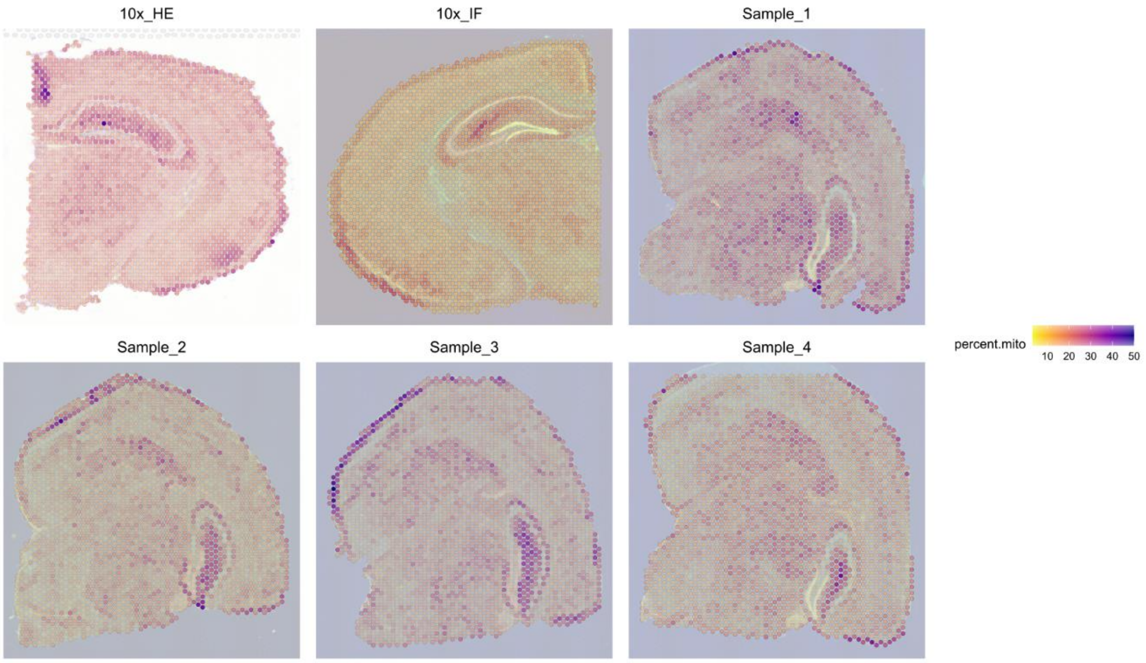

Supplement: Supplementary file 3 [file Data_Sheet_3.PDF]
