# Supplementary material for: Protecting RNA quality for spatial transcriptomics while improving immunofluorescent staining quality
Source: Front Neurosci. 2023 May 18;17:1198154. doi: 10.3389/fnins.2023.1198154 (PMC10234422; doi:10.3389/fnins.2023.1198154)

Supplementary Figure 4: Overview of immunostainings with antibodies that are compatible with our optimized protocol. Scale bar 1 mm. Scale bar overview 1 mm, scale bar magnifications 40µm.

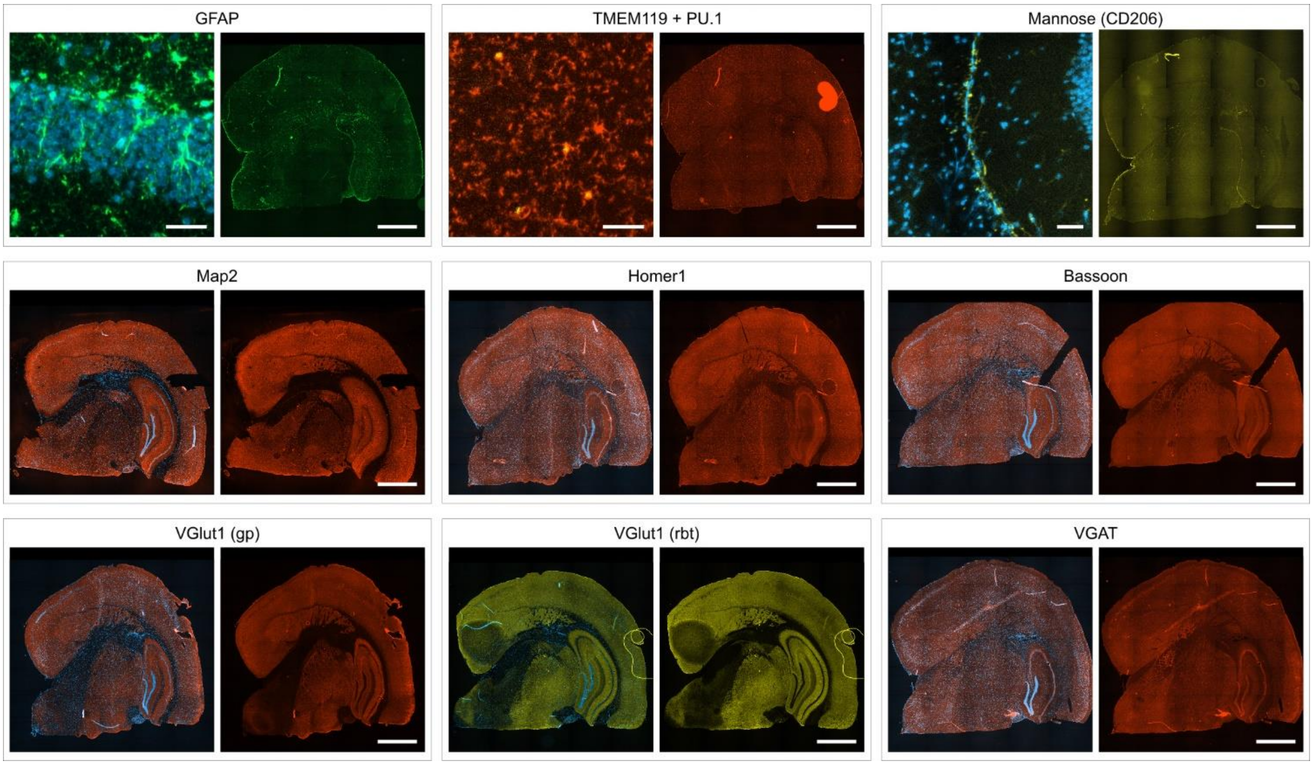

Supplement: Supplementary file 4 [file Data_Sheet_4.PDF]
