# Supplementary material for: Protecting RNA quality for spatial transcriptomics while improving immunofluorescent staining quality
Source: Front Neurosci. 2023 May 18;17:1198154. doi: 10.3389/fnins.2023.1198154 (PMC10234422; doi:10.3389/fnins.2023.1198154)

Supplementary Figure 5: Overview of immunostainings with antibodies that are not compatible with our optimized protocol.

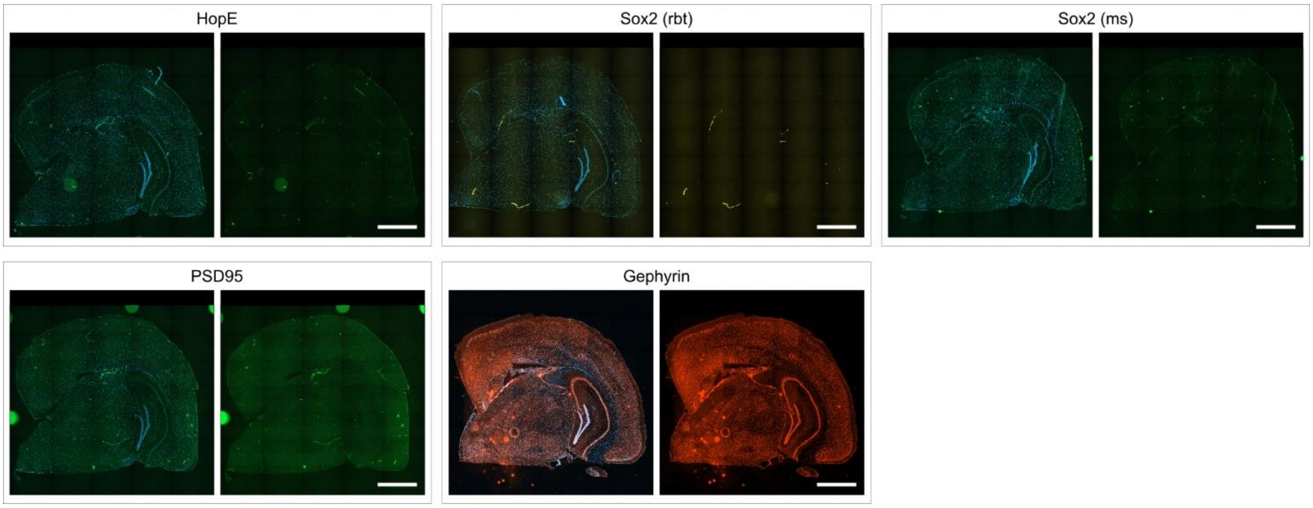

Supplement: Supplementary file 5 [file Data_Sheet_5.PDF]
