# Supplementary material for: Protecting RNA quality for spatial transcriptomics while improving immunofluorescent staining quality
Source: Front Neurosci. 2023 May 18;17:1198154. doi: 10.3389/fnins.2023.1198154 (PMC10234422; doi:10.3389/fnins.2023.1198154)
Supplement: Supplementary file 6 [file Data_Sheet_6.PDF]

Supplementary Table 1: Summary of sequencing metrics and quality control

	10x_HE	10x_IF	Sample_1	Sample_2	Sample_3	Sample_4
**Number of Reads**	312,266,904	157,247,248	236,522,674	262,005,857	26,312,569	221,525,677
**Valid Barcodes [%]**	97.4	97.3	96.9	96.8	96.9	96.9
**Valid UMIs [%]**	100.0	99.9	100.0	100.0	100.0	100.0
**Sequencing saturation [%]**	61.3	67.0	75.9	70.5	66.1	71.4
**Q30 Bases in Barcode [%]**	97.2	96.1	95.9	95.9	95.9	96.0
**Q30 Bases in RNA Read [%]**	94.5	94.0	94.3	94.7	94.5	94.3
**Q30 Bases in UMI [%]**	97.1	95.8	95.5	95.6	94.6	95.7
**Reads Mapped to Genome [%]**	94.9	94.6	80.3	73.4	75.4	78.3
**Reads Mapped Confidently to Genome [%]**	91.8	91.9	75.9	68.6	70.7	73.9
**Reads Mapped Confidently to Intergenic Regions [%]**	7.6	7.2	10.5	9.8	9.9	9.9
**Reads Mapped Confidently to Intronic Regions [%]**	2.5	3.1	9.5	9.2	9.1	8.4
**Reads Mapped Confidently to Exonic Regions [%]**	81.7	81.6	55.9	49.7	51.7	55.6
**Reads Mapped Confidently to Transcriptome [%]**	79.6	79.5	54.7	48.6	50.6	54.5
**Reads Mapped Antidense to Gene [%]**	1.1	1.2	0.5	0.4	0.4	0.5
**Number of Spots Unter Tissue**	2,702	2,807	2,438	2,432	2,484	2,509
**Fraction Reads in Spots Under Tissue [%]**	92.3	81.4	66.0	67.7	70.6	74.4
**Mean Reads per Spot**	115,569	56,020	97,015	107,733	105,928	88,292
**Mean Reads under Tissue per Spot**	103,089		61,073	73,511	72,693	62,675
**Median UMI counts per Spot**	28,941	11,048	7,994	9,982	12,222	9,613
**Median Genes per Spot**	6,018	4,221	3,321	3,766	4,229	3,702
**Total Genes Detected**	21,949	20,993	20,262	20,692	20,757	20,228
